# p53FamTaG: a database resource of human p53, p63 and p73 direct target genes combining *in silico *prediction and microarray data

**DOI:** 10.1186/1471-2105-8-S1-S20

**Published:** 2007-03-08

**Authors:** Elisabetta Sbisà, Domenico Catalano, Giorgio Grillo, Flavio Licciulli, Antonio Turi, Sabino Liuni, Graziano Pesole, Anna De Grassi, Mariano Francesco Caratozzolo, Anna Maria D'Erchia, Beatriz Navarro, Apollonia Tullo, Cecilia Saccone, Andreas Gisel

**Affiliations:** 1 Istituto di Tecnologie Biomediche-Sede di Bari, CNR, Via Amendola, 122/D 70126 Bari, Italy; 2 Dipartimento di Biochimica e Biologia Molecolare, "Ernesto Quagliariello", Università degli Studi di Bari, Via Orabona, 4, 70126 Bari, Italy

## Abstract

**Background:**

The p53 gene family consists of the three genes p53, p63 and p73, which have polyhedral non-overlapping functions in pivotal cellular processes such as DNA synthesis and repair, growth arrest, apoptosis, genome stability, angiogenesis, development and differentiation. These genes encode sequence-specific nuclear transcription factors that recognise the same responsive element (RE) in their target genes. Their inactivation or aberrant expression may determine tumour progression or developmental disease. The discovery of several protein isoforms with antagonistic roles, which are produced by the expression of different promoters and alternative splicing, widened the complexity of the scenario of the transcriptional network of the p53 family members. Therefore, the identification of the genes transactivated by p53 family members is crucial to understand the specific role for each gene in cell cycle regulation. We have combined a genome-wide computational search of p53 family REs and microarray analysis to identify new direct target genes. The huge amount of biological data produced has generated a critical need for bioinformatic tools able to manage and integrate such data and facilitate their retrieval and analysis.

**Description:**

We have developed the p53FamTaG database (p53 FAMily TArget Genes), a modular relational database, which contains p53 family direct target genes selected in the human genome searching for the presence of the REs and the expression profile of these target genes obtained by microarray experiments. p53FamTaG database also contains annotations of publicly available databases and links to other experimental data.

The genome-wide computational search of the REs was performed using PatSearch, a pattern-matching program implemented in the DNAfan tool. These data were integrated with the microarray results we produced from the overexpression of different isoforms of p53, p63 and p73 stably transfected in isogenic cell lines, allowing the comparative study of the transcriptional activity of all the proteins in the same cellular background.

p53FamTaG database is available free at

**Conclusion:**

p53FamTaG represents a unique integrated resource of human direct p53 family target genes that is extensively annotated and provides the users with an efficient query/retrieval system which displays the results of our microarray experiments and allows the export of RE sequences. The database was developed for supporting and integrating high-throughput *in silico* and experimental analyses and represents an important reference source of knowledge for research groups involved in the field of oncogenesis, apoptosis and cell cycle regulation.

## Background

The p53 gene family consists of the three genes p53, p63 and p73, each encoding for sequence-specific nuclear transcription factors. p53, the first member of the family to be identified, is involved in the maintenance of genome integrity by transactivating genes controlling cell growth and apoptosis in response to cellular stress signals [1,2]. p63 and p73 have been identified as p53 homologous genes which encode proteins showing significant similarity with p53 in the three main functional domains (transactivation, central DNA binding and tetramerization domains). They are able to transactivate some of the p53-responsive genes and to induce cell cycle arrest and apoptosis [3,4]. The p53 family members are able to bind the same responsive element (RE), initially identified for p53 [5,6] but with a degree of specificity for the target genes that is quantitatively distinct.

The p53 family members have a complex transcriptional pattern, encoding a great variety of protein isoforms. The p53, p73 and p63 genes through alternative splicing can produce different isoforms which differ in the C-terminal portion (α, β, γ for p53, α, β, γ, δ, ε, and ζ for p73 and α, β and γ for p63) [4,7-10]. Moreover, all three genes, through the use of two alternative promoters can encode two types of products, with or without the transactivation (TA) domain, the latter called ΔN isoforms. Accumulating evidence indicates that *in vivo *p53, p63 and p73 may be differentially regulated and can carry out specialized, non-overlapping functions. It has been suggested that, while the main role of p53 lies in the inhibition of tumour progression, p73 and p63 appear to be much more strongly involved in development and differentiation [11,12]. To understand the specific role that each member has acquired in cell cycle regulation, the identification of their target genes is crucial.

We combined a genome-wide computational search of p53 family REs and microarray analysis to identify new direct target genes of p53 family members. *In silico *analysis was performed using PatSearch, a pattern-matching program implemented in the DNAfan tool (DNA Feature Analyzer) developed in our Lab [13,14]. We optimised the original RE consensus on the basis of criteria deduced from experimental data to drastically reduce possible false positive hits. Since the presence of an RE in a gene does not imply by itself the binding and the transactivation activity of the transcription factor, we complemented the search of the putative p53 family transactivated genes with DNA microarray experiments using overexpression conditions. We investigated the transcription profiles of the potential target genes in stable isogenic cell lines (T-rex 293) overexpressing different members of the p53 family. This is the first study which reports microarray results of the overexpression activity of all the three family members in the same experimental conditions. Published data about identification of new p53, p63 or p73 target genes using microarray approach do not consider all three proteins in the same study. Moreover, the Authors, within all the up/down regulated genes of the array, only report the RE sequences of the few genes validated by direct binding assays [15-18]. Furthermore, these data are not collected in relational databases accessible through the web but in simple flat file collections with little additional information.

The p53FamTaG database (p53 FAMily TArget Genes) was developed to organize all the data produced by computational analysis and by microarray experiments. This database is the first and unique integrated resource of human direct p53 family target genes. It provides an efficient query/retrieval system, is integrated with other primary public data sources, and allows the export of the RE sequences to be used for further *in silico *and experimental analyses. Moreover, p53FamTaG contains the annotations of experimental data produced in other Labs. The structure and content of p53FamTaG will provide a valuable support and an important reference knowledge source for all research groups involved in the study of oncogenesis, apoptosis and cell cycle regulation.

## Construction and content

The p53FamTaG database has been developed to organize and integrate both the *in silico *and microarray data we produced with other experimental, bibliographic and computational annotation and to make them publicly available and retrievable as a web resource.

### Database schema

The p53FamTaG database is structured in a relational schema using the MySQL Database Management System. It was designed in a modular way so that data coming from computational, experimental analyses and public resources can be easily integrated and updated independently as and when needed.

The schema consists of three main modules: the "InSilicoData", the "RawData" and the "ExperimentalData" module.

The "InSilicoData" is the central module of the database which stores the information of the target hits (REs) obtained from *in silico *analysis, including: the RE sequences structured according to the pattern (decamers and spacers), the genomic coordinates, the genomic regions the REs were found in (intron, promoter, 5'UTR) and the gene names.

The "RawData" module is the repository section of the database that only stores the raw expression data and the experimental details of the microarray results as obtained by the Ab1700 (Applied Biosystems) microarray reader. This module will be important in a future version of the database when an automatic analysis of new microarray data will be implemented.

The "ExperimentalData" module contains the quantile normalized data, the gene expression values obtained from the application of statistical analysis on the microarray quantile normalized data (see the Data Generation chapter for further details), and the annotation of the genes spotted on the microarray provided by the manufacturer.

It is noteworthy to mention that the schema design links the "InSilicoData" and the "ExperimentalData" modules of the database allowing integration between heterogeneous sources (see Integration data paragraph).

### Data generation and database construction

#### *In silico *data

##### Definition of the optimal syntax pattern for the genome-wide search of human p53 responsive elements

The p53 Responsive Element (RE) is made up of two tandem repeated decamers complying with a specific consensus corresponding to the 5'-PuPuPuC(A/T)(T/A)GPyPyPy-3' sequence, allowing at most 3 mismatches. The decamers can be spaced by 0 to 13 nt and are generally located in the promoter region, or in the first introns of transactivated genes [6]. The same consensus is also recognised by p63 and p73. This consensus appears to be relatively non-stringent for transcription factors with such profound biological effects and produces huge numbers of hits located at any positions within the genome. In order to reduce the number of hits and to enhance the selectivity of the pattern searching analysis in a genome-wide search, we optimised the original RE consensus by introducing new criteria of stringency based on the comparative analysis of 109 human experimentally demonstrated REs contained in 83 target genes of the p53 family members. These genes have been demonstrated to be direct targets by different methodological approaches (measurements of transactivation activity, EMSA, Chromatin immuno-precipitation) (for references, the book-buttons in the database provide the link to PubMed).

To highlight the structural features of these 109 REs we considered: 1) the conservation of each specific position in the two decamers, 2) the spacer length between the decamers, 3) the position of the REs with respect to the transcription start site (TSS), and 4) the presence of additional decamers.

To compute the degree of conservation of each specific position in the two decamers we performed a multiple alignment of these sequences (excluding the spacers between the decamers) and applied the Consensus program of RSA-Tools (Regulatory Sequence Analysis Tools) which produces a probabilistic description of the sequences based on PWMs (Position Weight Matrices).

Interestingly, we found that 95% of the p53 REs have up to 8 spacer bases (sb). In particular, 62% have up to 2 sb and no RE has 12 or 13 sb. The analysis of the RE location relative to the TSS of the target genes as mapped in ENSEMBL revealed that: 50 out of 51 known REs identified in the promoter regions, were located within 3500 bp upstream of the TSS, and 52 out of 58 known REs identified in the introns were present within 20,000 bases downstream of the TSS.

To further increase the accuracy of RE prediction we also searched for additional decamers. Indeed, other studies have reported that the canonical p53 target sequence may include additional decamers where up to 3 mismatches are allowed [5]. By using the PatSearch program [14] to search for a pattern following the criteria mentioned above including a third decamer, we found an additional third decamer very often not observed by the Authors, within all the 83 experimentally demonstrated p53 target genes.

**Figure 1:**
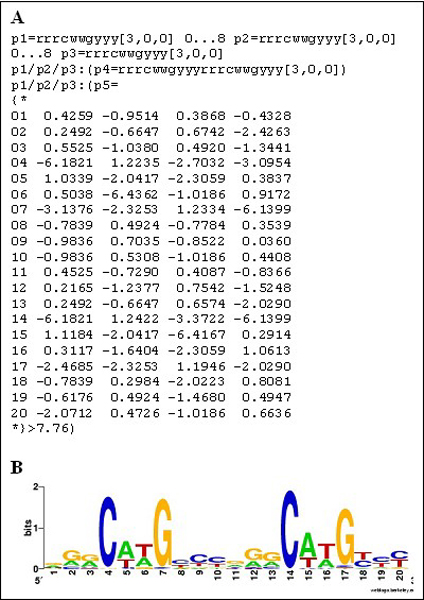
**PatSearch syntax and Sequence logo of the p53RE**. A) PatSearch syntax for the *in silico *search of the p53 gene family REs including the two decamer PWM. B) Sequence logo of the p53RE derived from the PWM.

In conclusion, on the basis of experimental data we limited the search analysis for p53 gene family REs, to a region ranging from -3500 (in the promoter region) to +20000 (in intron regions) with respect to the gene TSS. In this search we included the 5'UTR and excluded the annotated coding exons. We modified the consensus syntax including 3 decamers spaced by 0 to 8 bases, allowing up to 3 total mismatches in two adjacent decamers, tolerating up to 3 mismatches in the third decamer. A post-processing phase of PatSearch matching the inferred PWM (Figure 1A) was also carried out (see below). The sequence logo of the p53RE derived from the PWM is shown in Figure 1B.

##### Search of the p53 responsive elements in the human genome

The computational analysis to identify the human REs was performed using the PatSearch algorithm implemented in the DNAfan tool [13,14]. DNAfan filter facilities allow the user to analyze specific genome regions (e.g. promoter regions, 5'UTR, introns, etc.). The program therefore automatically generates on feature key annotation, a specific sequence dataset spanning a given feature key on which the desired analysis program, in our case PatSearch, is executed. PatSearch is particularly suitable for searching sequence data for the presence of complex oligonucleotide patterns, the structure of which was derived from experimental characterization of functional elements. Using the syntax pattern previously described (Figure 1A), we performed a genome-wide search in the ENSEMBL database (release 34) and found 63384 REs in 18.110 genes, after redundancy cleaning based on their absolute genome coordinates.

The relevant information contained in DNAFan output, including the sequence of the REs, the pattern structure according to the specific syntax (decamers, spacers), and a set of genomic information such as ENSEMBL gene ID (ENSG), gene name (HUGO nomenclature), chromosome, strand direction and absolute start site, was uploaded into the "InSilicoData" tables of the database using a perl script.

**Figure 2:**
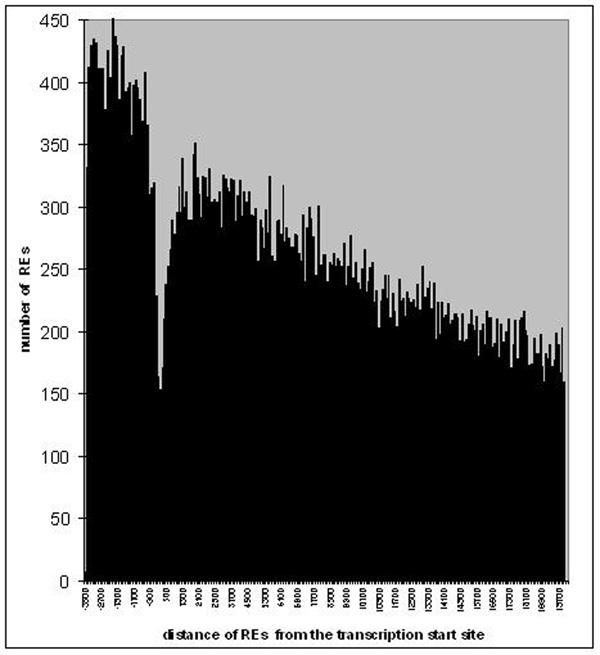
**Distribution of the REs**. Distribution of the database set of 63890 REs, over a 100 nt window, along the human genomic sequence from -3500 nt upstream to 20000 nt downstream of the gene transcription start site (TSS). There is an evident significant reduction in frequency of the REs in the region immediately downstream of the TSS corresponding to the 5'UTRs.

Figure 2 reports the genome wide description of the distribution of REs in non-translated genomic sequences (promoters, 5'UTRs and introns). The highest density of REs is concentrated in the TSS upstream regions (promoters) and slightly decreases along the intron sequences, proportionally to the downstream distance from the gene TSS. Interestingly, a significant reduction in RE frequency is evident in the region immediately downstream of the TSS corresponding to the 5'UTRs. This distribution trend is coherent with the localization of known experimentally validated REs, further supporting the validity of our *in silico *search criterion.

#### Microarray data

The *in silico *analysis was complemented by an experimental microarray approach. In order to examine the consequences of the overexpression of different members of the p53 family on their target genes under comparable conditions, we created human embryonic kidney Flp-In T-Rex-293 stable isogenic cell lines expressing the different genes under the control of tetracycline inducible promoter. As a control we created a stable cell line containing CAT (Chloramphenicolo acetyl transferase) cDNA. For microarray hybridization, mRNA was extracted from cell lines at 6 h, 24 h after induction of the different proteins and 20 μg were labeled by retrotranscription and hybridised to the microarray (Applied Biosystems Human Genome Survey Microarray V1.0 containing 33202 probes for the interrogation of 27686 genes) according to the protocols supplied by the manufacturer (Applied Biosystems).

The resulting data set consists of 6 different experimental conditions (Flp-In T-Rex-293 cells stably transfected with: p53, p53R175H, TAp63α, ΔNp63α, TAp73α, TAp73β) analysed in two technical replicas probed at two different time points (6 and 24 h) and two technical replicas of the control experiment. Therefore we processed the data generated from 26 microarrays.

The expression values (raw data) including statistical quality measurements for each microarray were obtained as exports from the Ab1700 (Applied Biosystems) software. Quantile normalization, using the statistical software package R, were performed on the whole expression data set (~33000 probeID × 26 microarrays) to obtain entirely comparable data within and between microarrays. The raw data including the statistical quality measurements were uploaded into the "RawData" tables of the database using a perl script saving the raw signal, assay-normalised signal, signal to noise (S/N), and the flag value. For every array design we also had to parse and upload the annotation of the corresponding genes spotted on the microarray provided by the manufacturer. The algorithm's quality metrics flags and the ratio of S/N were used to quantify probe quality and "detectability" in each sample-control comparison (1700 Chemiluminescent Microarray Analyzer Users Guide). Only probes with <5000 flags in both replicas and S/N ≥ 3 in at least one replica in both sample and control were positively filtered and used for successive statistical analysis.

In the subsequent step of data analysis, a tool based on a Bayesian statistical framework, CyberT, was applied to compare normalized expression values of filtered probes between each sample and control. The tool allows the identification of differentially expressed genes with a higher level of confidence than a simple t-test, when only few experimental replicates are available, and implements a posterior probability of differential expression-based method (PPDE(<p)) for calculating the global false-positive and false-negative levels [19].

Microarray probes were classified as differentially expressed, in each sample-control comparison, if PPDE(<p) >0.995 (posterior probability of differentially expressed genes). This threshold corresponds to log transformed Bayesian p-value thresholds, which are different in each experiment, but always lower than 0.0032. For these probes, fold change was reported as S/C (when S>C) or -C/S (when C>S) where S is the sample expression value and C is the control expression value. Probes that were not significantly up or down regulated and those negatively selected in the filtering step were also identified and reported without fold change indication.

The quantile normalized expression data used for the statistical analysis and the Cyber-T results (fold-change, Bayes.lnp and the PPDE (<p)) were uploaded into the "ExperimentalData" tables using a perl script. Cyber-T results can be easily recalibrated by changing the cut-off values.

### Data integration and annotation

Due to the heterogeneity of the data sources we used to produce the *in silico *and experimental results and the absence of a standardized annotation between them, the most complex task was linking the modules. In particular, the *in silico *data were referred to ENSEMBL annotation (loaded in the "InSilicoData" module) and the gene expression data were referred to the Ab1700 (Applied Biosystems) microarray chip annotation extracted from the CELERA Panther^© ^system (loaded in the "ExperimentalData" module). To link these two data sets, we extracted the RefSeq accession number and the HUGO gene names from the microarray annotation and linked them to the ProbeID which is the unique identifier of the probe on the microarray chip. In parallel, for the ENSG of each gene containing the RE, the RefSeq accession number and the HUGO gene names were extracted from ENSEMBL. A simple link was established by the comparison and a successive association, implemented by iterative SQL statement, between REs and microarray spots (ProbeID) having the same Gene name and Refseq accession number in the two data sets. In this way we established 17644 links between the *in silico* data and the spotted genes on the microarray. However, only 4874 genes, which contain the REs, have a significant microarray result in at least one experimental condition.

Each gene stored in the database has extensive annotation extracted from different public resources such as: ENSEMBL Gene ID, gene name and aliases, RefSeq accession number, Celera gene ProbeID.

The p53FamTaG database also annotates other experimental data produced in different labs. In particular, the 83 genes stored in the database that were experimentally demonstrated as direct target genes by other Authors, are linked through PubMed to the abstract of the paper reporting the data. Moreover, 341 p53 high-confidence binding loci obtained using the genome wide ChiP-PET approach reported in a recent paper [20] have been annotated in the database and linked to PubMed and to UCSC, where the annotation can be found in the ENCODE Chromatin Immunoprecipitation tracks under the p53 ChiP-PET analysis (GIS p53 5 FU HCT116 Track Settings).

A summary of the database content is reported in Table 1.

## Utility

The complexity of the schema and the quantity of information stored in the database, represented a challenge in terms of visualisation and navigation.

The p53FamTaG database is freely available through a web-application developed in PHP (Seagull framework) and can be queried in a user-friendly manner through a graphical user interface (GUI). Wizard-like steps were developed to drive the user into the navigation.

A horizontal menu bar provides links to the Home page, Search form and on-line users' Manual.

**Figure 3:**
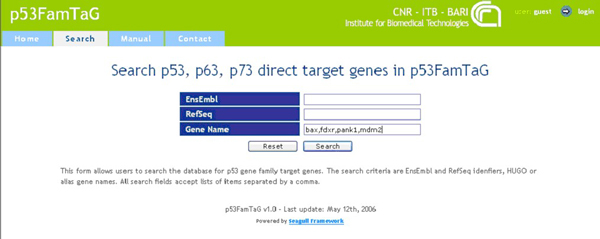
**Database query form**. This form allows the users to search the database for p53 gene family target genes. The search criteria are ENSEMBL and RefSeq idenfiers, HUGO or alias gene names. All search fields accept lists of items separated by a comma and execute a search in OR mode. The database has been queried for the BAX, FDXR, MDM2 and PANK1 genes.

The 'Search' form (Figure 3) allows the users to search the database for p53 gene family target genes. The search criteria are ENSEMBL and RefSeq idenfiers, HUGO or alias gene names. All search fields accept lists of items separated by a comma and execute the search in OR mode (case study: bax, fdxr, mdm2, pank1).

**Figure 4:**
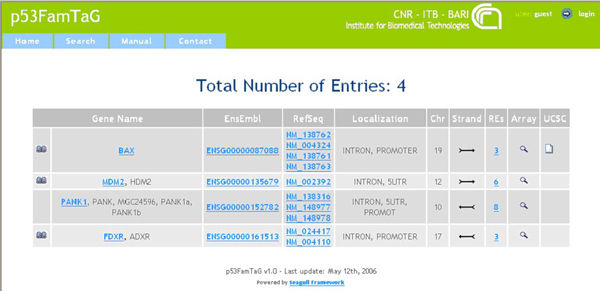
**Query result page**. Results are displayed as a table summarising information on retrieved entry. The Gene Name, ENSG entry and NM entry are linked to the HGNC, ENSEMBL and RefSeq databases respectively. The clickable book provides access to PubMed for the transactivated genes (BAX, FDXR, MDM2) and for ChIP-PET high confidence binding loci (BAX) references. The Array button, when pressed, shows the microarray results (Fig. 7), the UCSC button activates the link to the annotation in UCSC of ChIP-PET binding loci (BAX). The alias gene name, the number of REs, the gene region localization, the strand orientation, and the chromosome also are reported. The REs button shows the number of the hits and, when pressed, more detailed information (Fig. 5).

**Figure 5:**
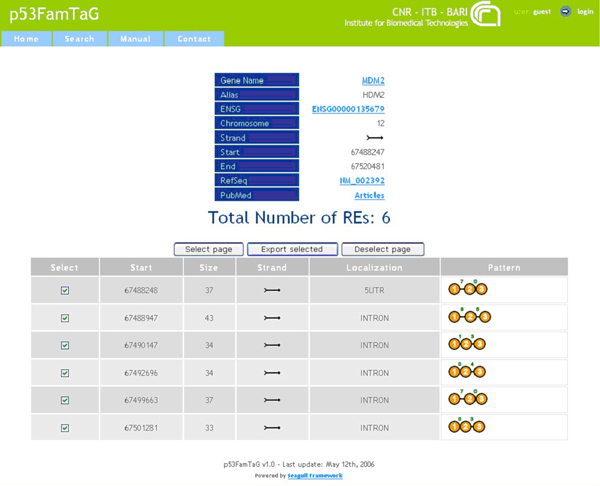
**Details of the REs of one ENSG**. The table shows the gene information at the top and below the number and list of hits (REs) reporting the chromosomal position, size, strand orientation and the gene region localization, as well as a graphical representation of the target sequence depicting the pattern (decamers and spacers). The Select/Deselect buttons allow the selection of RE sequences for export (Fig. 6).

**Figure 6:**
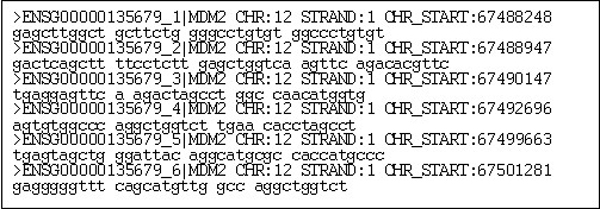
**RE Sequence export form**. Export form of the selected RE sequences in FASTA format, with the indication of the decamers and spacers, and the exact genomic coordinates.

The query report (Figure 4) lists the matching database records ordered by ENSG. It reports the gene and alias name, ENSEMBL stable gene ID (ENSG), RefSeq accession number, the gene region where the RE was found (intron, 5'UTR, promoter), the chromosome, the strand orientation and the number of possible target sites (REs column). The gene name, the ENSEMBL ENSG and the RefSeq ID are hyperlinks to the HGNC, ENSEMBL and RefSeq databases, respectively. A magnifying glass-button, in the array column, provides a link to microarray data, while the book-button in the Gene name column allows the PubMed reference to be consulted for the experimentally demonstrated target genes. The UCSC-button in the UCSC column links genes identified by ChIP-PET analysis to the UCSC database. Moreover, a hyperlink of the hit number, in the REs column, leads to more detailed information about the different hit/REs including the genomic coordinates, the size and a graphical representation of the target sequence depicting the pattern (decamers and spacers) (Figure 5). From this page it is possible to easily export the selected RE sequences in FASTA format, with the indication of the decamers and spacers, and the exact genomic coordinates (Figure 6).

**Figure 7:**
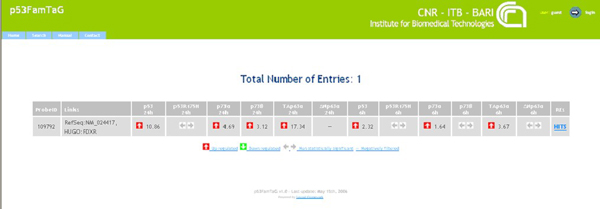
**Details of Microarray results**. The Microarray data were obtained by the overexpression of wild type p53, p53 mutated form p53R175H, TAp73α, TAp73β, TAp63α, ΔNp63α, at 24 h and 6 h. The table shows the Celera Probe ID, detecting the gene on the array, and the fold change values of the up/down regulated gene in each cell line. The symbols on the bottom link to the Manual for details of expression data analysis.

When the ENSG of interest has a result in the microarray data, a link leads the user to the expression data of the gene. The new page visualises the expression profile of this gene (for each Celera probe ID representing a gene on the array) in all the stable isogenic cell lines (Figure 7) with graphical icons. The results of the gene expression are indicated as "Up regulated", "Down regulated", "Not statistically significant" or "Negatively filtered". "Up regulated" is the fold change value reported as S/C (when S>C) while "Down regulated" is the fold change value reported as -C/S (when C>S), where S is the expression value of the gene in the Sample, and C is the expression value of the gene in the Control. "Not statistically significant" indicates expression values with PPDE(<p) <0.995 and "Negatively filtered" indicates expression values filtered out by quality control (flag >5000 and signal to noise S/N < 3).

In conclusion, the user can query the database to find out whether or not a gene of interest contains a p53 gene family RE and how this gene is expressed under overexpression of the three members of the p53 gene family in 293 T-rex cells.

## Discussion

The recent explosion of "knowledge production" due to the completion of human genome sequences and to the availability of high-throughput technology (such as microarray, ChIP-PET, Chip-on-Chip) has generated a critical need for bioinformatic instruments able to manage the huge amount of biological data produced and to facilitate their retrieval and analysis. Moreover, the integration of data from different resources, such as *in silico *analysis, experimental data and biological databases, is crucial to exploit the large amount of information available and to focus on the discovery of the functional role of genes, and their expression regulation.

The p53FamTaG database is a comprehensive and unique resource of genome-wide search of human p53, p63, p73 direct target genes combining the *in silico *prediction of their p53 REs, with the transcriptional profiles of these target genes in isogenic cell lines over-expressing different members of the p53 family. The dissection of the transcriptional targets of p53 gene family members, which recognise the same RE, is a challenge in cancer research. p53 is mutated in over 50% of all cancers and in the remaining cases its pathway may also be affected. The involvement of p73 and p63 in tumour development is much less well-established. Multiple isoforms of p63 and p73 have been characterised and emerging evidence suggests that some of the roles played by the TAp63 and TAp73 isoforms overlap those of p53, whereas their ΔN variants have an opposite effect or even an oncogenic role in cancer progression.

To identify putative direct target genes of the p53 gene family, we performed an *in silico *genome-wide search of the p53 REs in specific regions of the human genes (promoter region, introns, 5'UTRs), using criteria defined on the structure of 109 REs of 83 human experimentally demonstrated target genes. Through this *in silico* search we selected 18110 human genes containing the REs as potential p53 family direct target genes.

We complemented and validated the *in silico *results with the study of the expression profile of these potential direct target genes using the microarray approach. To this purpose we generated stable transfected cell lines integrating the expression constructs of p53, TAp63α, ΔNp63α, TAp73α, TAp73β and the p53R175H mutated isoform in the same genomic locus. This allowed us to examine, in the same cellular context, the effects of the overexpression of p53 family members on the expression profile of the *in silico *detected direct target genes.

In recent years, distinct studies have aimed to identify p53, p63 and p73 regulated genes using DNA microarray approaches. However, all the three proteins have not be analysed so far in the same study and considering the heterogeneity of experimental and genetic backgrounds used (cellular stressors, cell lines, etc), it is very difficult, if not impossible, to compare the expression profiles of those independent studies to identify common and non-common target genes and whether those genes are direct or indirect targets [15-18]. A further drawback of these results is that they exists only as simple flat files, poorly annotated and they are not collected in relational databases publicly available through the web.

p53FamTaG database was designed to store the information of the *in silico *and microarray approaches with links between the two data sets and to the most accredited databases world-wide. Through a user-friendly graphical interface, it is possible to query this complex information in a few seconds. For each gene containing the RE, the database provides the gene name (HUGO), the alias name, the ENSEMBL stable gene ID and RefSeq ID, the chromosome, the RE structure (decamers, spacers, length, sequence), the RE chromosomal position and gene region localization (promoter, 5'UTR, intron) and the microarray results. Moreover, the database provides the hyperlink to PubMed for the experimentally demonstrated target genes.

One particularly noteworthy feature of the database is the possibility to export the sequences of the REs including full information in FASTA format, which is not possible from any other public resource. The availability of the RE sequences of potential target genes which appear to be up or down regulated in our microarray experiments, allows to guide experimental approaches (such as PCR amplification of REs and cloning for luciferase, EMSA, Chromatin immuno-precipitation assays) to demonstrate the binding of the p53 family member to the RE (manuscript in preparation). Furthermore, these results may lead to refining the specific RE for each p53 family member and finally to identifying common transcription binding site frameworks by applying algorithms.

An additional significant feature of p53FamTaG database is the annotation of 83 experimentally demonstrated direct target genes integrated with the microarray data produced in our Lab. These target genes are often only validated for one of the members or isoforms of the p53 family members. Our data set now allows the user to observe such a target gene under the overexpression of the three p53 family members under identical experimental conditions and to understand the involvement of each member in the modulation of this target gene.

A map of 542 human p53 high-confidence binding loci, obtained by ChIP (chromatin immunoprecipitation)-PET (paired-end ditag) approach, has recently been published [20]. The PET sequences are derived from about 66,000 individual p53 ChIP fragment sequences using human HCT116 colorectal cancer cells treated with 5-fluorouracil for 6 h, conditions known to activate p53 expression. The gene name, the sequence and the chromosomal localization of the 542 binding loci PET clusters are available in the UCSC database. However, the REs are not indicated and therefore not suitable for further studies. Out of the 542 loci, only 381 corresponded to known genes with the others referring to sequences lacking gene names, to cDNA clones or to hypothetical proteins. We queried our p53FamTaG database by using the list of these 381 gene names and we found that 341 genes are present in our database, showing that our pure *in silico *approach found most of these experimentally selected RE. Moreover, for 205 of these target genes our database reports the microarray results, making available for these genes, studied only for p53, also the transcriptional effect of p53R175H, TAp63α, ΔNp63α, TAp73α and TAp73β under our experimental conditions.

The availability of a database like p53FamTaG able to integrate, retrieve and display this precious information also led us to find a way to include the ChIP-PET data through a link to the UCSC database.

It should be considered that high throughput technology (microarray, ChIP-PET, Chip-on-Chip) has the advantage of producing and analysing large quantities of biological data, yet it is based on robust experimental methodology (which also has a substantial cost) and statistical analysis. However, each experiment in any case remains referred to specific conditions (type of inductors, cellular stressors, cell line), and represents an initial framework for further experimental validation more focused on particular cellular conditions. In particular, in gene expression studies, among the regulatory elements (p53 family members REs in our case) spread throughout the genome, only some are involved in specific conditions following binding dynamics or tissue specificity of the transcriptional network. For example, out of the 83 direct target genes that have been experimentally demonstrated and are annotated in our database, only 15 genes were identified in the global map study with the ChIP-PET approach reporting 542 p53 binding sites.

With the global *in silico *search the p53FamTaG database is able to collect all the information and put it into a holistic picture creating new knowledge that would not be possible on a single data set. Therefore, p53FamTaG represents a powerful resource and may be considered a basic repository colligating existing information for researchers in this field. The database content is reported in Table 1. The validity of the *in silico *data we obtained by applying DNAfan and in particular the consistence of the criteria we established for the genome-wide RE search (stringency and complexity of the search pattern) were supported by the positive selection of 83 experimentally demonstrated p53 target genes and of 341 ChiP-PET high-confidence binding loci reported in literature. Moreover, the majority of the published target genes present in p53FamTaG have a statistically significant change of expression in at least 1 sample of our experimental conditions. These results strongly validate the complementary approach between the *in silico* search and the microarray experiments.

The important features of the structure of the database are: 1) its design in modules so that, with the perspective of data integration, it can be easily extended to host additional data such as new experimental data (results from microarray or real-time PCR approaches), and data from the literature, making the new data available in context with the target sequence analysis; 2) the design can be mirrored for example for the identification and collection of the REs in other organisms or for the *in silico *identification of other transcription factor binding sites.

## Conclusion

The p53FamTaG database is the first and only available publicly resource developed for supporting and integrating high-throughput *in silico* and experimental analyses of p53 family target genes and represents an important reference source for research groups involved in the fields of oncogenesis, apoptosis and cell cycle regulation. p53FamTaG includes a wide number of human genes (18,110) containing the REs obtained from a large-scale human genome analysis, based on criteria derived from experimental conditions. Information stored in the database can be easily queried by searching for a particular gene of interest or for a dataset of genes produced by other methodologies (e.g. a list of genes up/down regulated in other microarray studies) for the identification of novel direct p53 family target genes.

This strategy of combining *in silico *and experimental approaches and the possibility to compare different methodological approaches as well as the effect of the wild-type and oncogenic isoforms may allow the reconstruction of the comprehensive p53 family downstream network and will be of great help in elucidating the particular defects in the p53 family response associated with human cancers thus generating new tools for diagnostic, medical and pharmacological applications.

## Availability and requirements

p53FamTaG database is publicly available at [21]. The e_mail bigstaff@ba.itb.cnr.it may be used for questions, comments, suggestions and corrections.

## Authors' contributions

The Authors contributed to the work according to their expertise in the data generation and database construction. FL and AG designed and implemented the relational scheme of the database. FL, DC and AG designed and implemented the data upload and annotation procedures. GG developed an improved and customized version of the DNAFan tool. ATuri developed the Web application, including: GUI interface, query and data extraction system, SVG graphical visualization modules. GP, SL, BN and ES participated in the definition and in the search of the REs. DC, GG and ADG performed the data quality control. ATullo, AMDE, BN and MC constructed the isogenic cell lines, performed the microarray experiments and produced the biological data. AG and ADG performed the statistical analysis of the microarray data. GP and CS provided precious suggestions on features of the database. ES coordinated and supervised the whole project together with AG.

## Figures and Tables

**Table 1:** Database Content

Genes containing REs	18110
REs in promoters	13649
REs in 5'UTRs	563
REs in introns	49678
Microarray data of genes containing the RE	4874
Experimentally demonstrated target genes	83
ChiP-PET high-confidence binding loci	341
